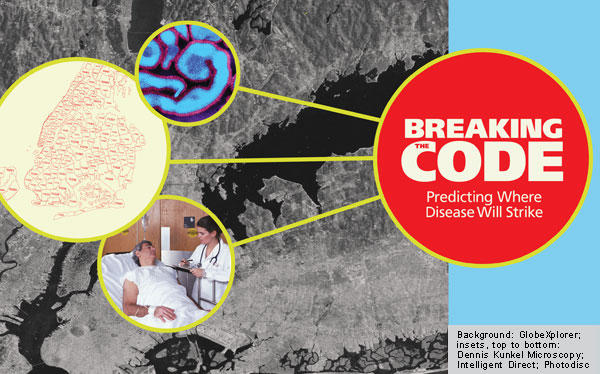# BREAKING THE CODE: Predicting Where Disease Will Strike

**DOI:** 10.1289/ehp.113-a610

**Published:** 2005-09

**Authors:** Tim Lougheed

Health authorities today enjoy an embarrassment of riches when it comes to data on disease incidence, thanks to the rapidly increasing use of electronic record keeping at hospitals, clinics, and related businesses such as pharmacies. Where significant numbers of paper files once required days or weeks to process for survey purposes, much larger volumes of material on patient conditions can today be assembled in a matter of hours. This growing abundance of information has raised expectations about the prospect of identifying local outbreaks of disease at ever earlier stages so that such outbreaks may be addressed and contained as soon as possible—expectations that are perhaps more easily raised than met, given statistical realities.

One of the most recent strategies for harnessing these data is offering some promising results, however. A paper that appeared in the March 2005 edition of *PLoS Medicine* outlines a new methodology that requires only that researchers know the actual number of disease cases occurring in a given area over a given period of time. The resulting statistical model makes only the most limited number of assumptions surrounding the emergence of a disease, while attempting to compensate for naturally occurring temporal and geographical variations in disease reporting.

This offers greater detail than one-dimensional statistical methods, which track disease outbreaks in either purely temporal or purely geographic terms. Above all, the new model eliminates the need for information about the local population and its relative risk for disease—for example, whether a neighborhood contains a higher-than-average proportion of groups, such as infants or the elderly, who may be prone to specific ailments.

## A Window on Disease

Underlying the work presented in *PLoS Medicine* is the principle of the scan statistic, which offers a probability of an excessive number of case reports appearing within a narrowly defined space and time, as compared with a probability determined from information collected in a larger region or over a longer period. Scan statistics aren’t new, but the *PLoS Medicine* paper adds a twist: the calculation of probabilities for disease outbreak within various samples of space and time. In the case of purely geographic surveillance, sudden highly localized outbreaks may be hidden in the data that have been aggregated for a region, whereas such events are more likely to be revealed once a temporal dimension has been incorporated. Space–time permutation scan statistics could therefore become a preferred way of representing the occurrence of diseases such as cancer, where the number of actual and expected reports are counted within particular “windows.”

These windows can be visualized as a set of thousands or even millions of overlapping “cylinders” within the geographic area in question, each varying in the amount of territory and length of time it covers. Harvard University Department of Ambulatory Care and Prevention associate professor Martin Kulldorff, one of the paper’s authors, uses the cylinder as a way of visualizing the sampling of data in three dimensions, with the x and y axes representing the geographic area being surveyed and a z axis representing time. As time passes, subsequent samples are added atop previous ones, and the cylinder thus grows in height. Mathematically, each cylinder uses a likelihood function to compare its expected and observed numbers of cases, making it possible to single out locations and days in which the latter number was unexpectedly high.

The new model was tested in conjunction with the New York City Department of Health and Mental Hygiene, which has been among the leading agencies collecting the data necessary to gauge the spread of disease. In the late 1990s, the city launched a dedicated program of syndromic surveillance, tracking ambulance reports, emergency room visits, and pharmacy sales—all with the aim of spotting anomalous clusters of cases that could signal a disease outbreak.

Kulldorff says the researchers took several steps to manage the computational burden of this exercise. The circular cylinder base was in turn one of several combinations of 183 New York City zip codes with a radius of zero to 5 kilometers. Each of the cylinders the researchers defined was seven “days” high (the team reasoned that if an outbreak has existed for more than a week, it will likely have already been picked up by clinicians or laboratories).

But while the geographic area covered by each cylinder remained the same, the specific days changed. For example, over the course of a month running from day 1 to day 30, the first statistical analysis would take place on a cylinder with a height defined by data from day 1 to day 7, the next would take place on a cylinder with a height defined by data from day 2 to day 8, and so on. This moving window makes it possible to look for changes taking place in a strictly defined time and space.

Thus, the team could, for example, catch a disease outbreak that began emerging on day 7, something that health authorities might not otherwise have identified for many more days. This early signal would prompt officials to check out the situation sooner and perhaps contain any outbreaks more successfully. To keep such signals in perspective, the statistical analyses also refer to the previous 30 days, so that any longer-term trends or variations could be compared with what has been seen in the seven-day window.

## Evaluating the Approach

The evaluation of this approach began by focusing on historical reports of diarrhea taken from emergency department data collected daily by the Department of Health and Mental Hygiene between November 2001 and November 2002. Files categorized individual cases according to nonspecific conditions or symptoms, such as “diarrhea” or “flu-like,” and included the zip code of patients’ homes and where they were treated.

Four of the five most statistically significant groupings of data produced by the model correlated to citywide outbreaks of rotavirus, norovirus, and influenza during the study period, meaning this information would have given early warning of these outbreaks had it been available at the time. And while the system did generate some signals with no correlating outbreak, these were relatively few.

One problem the team had to address was the fact that people work during specific times of the week, and that clinics or pharmacies may operate only between set hours. This creates variations that could create false signals, such as high sales on Sunday for drugs from a pharmacy that is open when most others in the area are closed. Such distortions were avoided by taking each day of the week into account when calculating the random probabilities of disease outbreaks. If data appeared to show a clustering of disease on a Sunday, for example, the probability of that finding was assessed relative to other Sundays, rather than to any given day of the week.

More problematic for many systems is inconsistent reporting of data across locations and days. “These are challenges in the field as a whole,” says Kulldorff, noting that many sophisticated electronic systems experience reporting lags. “One of those challenges is the timeliness of the report, in the sense that sometimes there are partly missing data. You maybe only get ninety percent of the data, and the rest doesn’t come in until a few days later.” Nevertheless, he regards these initial results from the space–time permutation scan statistic to be promising enough to warrant much more intensive study.

Kulldorff would like to hone the model by evaluating its performance with other sources of information. “It’s not clear what data sets are actually the best to use for infectious disease outbreaks—emergency department visits, ambulatory care visits, laboratory test results, and so on,” he says. Nor is it clear that the model should be limited to tracking natural disease outbreaks. He suggests that it could be successfully applied to other public health phenomena, such as the spread of antibiotic-resistant bacteria, as well as in completely distinct fields such as criminology, ecology, or engineering.

## Software Solution

In order to promote these broader applications, the model has been incorporated into SaTScan™, software Kulldorff has been developing since before he began working with the Department of Health and Mental Hygiene a few years ago. The program can be downloaded for free at http://www.satscan.org.

SaTScan’s purpose is to identify anomalous clusters of data that can be related to disease—that is, aggregations that are statistically distinct from the regular variations of health information that are being assembled from day to day in a particular area. Such anomalies can take a temporal form (cropping up in a short period) or a geographic form (occurring in a small region).

One of Kulldorff’s departmental colleagues at Harvard has reviewed the use of SaTScan for assessing disease outbreaks, including ones that could be the result of deliberate actions. Katherine Yih was part of a team working with the National Bioterrorism Syndromic Surveillance Demonstration Program, an initiative mounted by the Centers for Disease Control and Prevention (CDC) in collaboration with health care organizations covering more than 20 million people across various states. The goal of this program is to use data from health plans and practice groups to detect localized outbreaks and facilitate rapid public health follow-up.

Program researchers examined ambulatory patient records—reflecting medical care that was provided in a clinical setting rather than a hospital—which were organized according to zip codes as in the New York City project. The records were assembled on a 24-hour cycle; each night the system would look for clusters of illness that might signal an outbreak of statistical significance, so that an alert could be issued to officials in the affected area.

In a paper published in the 24 September 2004 issue of the CDC’s *Morbidity and Mortality Weekly Report*, Yih and her colleagues reported detecting unusual respiratory illness clusters in Colorado, Texas, and Massachusetts that were associated with severe influenza outbreaks. They are now turning their attention to evaluating the system’s ability to detect naturally occurring outbreaks of gastrointestinal illness, using data taken from known outbreaks that were identified by the Minnesota Department of Health. They are also carrying out simulations to determine if the system would be sensitive to acts of bioterrorism.

Yih endorses the approach, although she notes a few limitations. “The data are collected for other purposes,” she says. For instance, some of her most recent work draws on information from a Minnesota-based group that uses a triage nurse to provide health care information by telephone. The priorities of organizations operating such services start with trying to relate a patient’s self-reported symptoms to a readily identifiable disease group, rather than just recording the symptoms by themselves. That makes sense in terms of providing immediate advice to a caller, but it means researchers like Yih may have a much harder time going through the organization’s recorded data to find the symptoms that were being reported.

“The appeal of it is that you don’t have to request that a bunch of people at these health care organizations spend time entering data into your nice survey format or data format. They enter the data in the course of their routine patient care,” she says. By the same token, she adds, these data may not take a form or be timely enough to be optimal for identifying disease outbreaks. Nor is it always certain that the data capture a proportion of the population sufficient to justify an alert. For example, as little as 5–15% of the people in a metropolitan area might be covered by a health plan that may be supplying the data.

Kulldorff and his colleagues cite a complementary issue that affects their own disease monitoring strategy, which detects specific outbreaks at a highly localized level rather than simultaneous outbreaks taking place over a broad surveillance area. If an infectious agent is transmitted on the subway, for example, the people affected will not necessarily live anywhere near one another, nor are they likely to wind up in the same emergency room.

Moreover, their method counts on a disease having symptoms severe enough to send someone to the emergency room, making it harder to detect small outbreaks of less serious ailments. For this reason, they do not offer this approach as an exclusive choice. Instead, they suggest that efficient and comprehensive surveillance should be based on the use of different detection systems, each with its own strengths and weaknesses.

Yih recommends ongoing evaluations of SaTScan and similar systems to determine whether the expenditure of resources for disease data monitoring programs—as well as resources that public health officials would put into following up on the warning signals generated by those programs—would be justified. Ideally those signals should make life easier for public officials, ensuring that their efforts will be all the more effective.

Such assurance is the real service that systems such as scan statistics can offer to these officials, says Emory University biostatistician Lance Waller, who has critiqued the methodology behind disease surveillance models. “These databases that were collected separately and sat on their own computers before, can now be put together,” he says. “That’s a new thing for public health workers—to be able to get multiple health records from multiple hospitals, pharmacy sales from drug stores. These kinds of methods are good tools in a toolbox.”

New York City seems to think the system is a good thing. Since 2003, the scanning software has been applied daily using the city’s surveillance system, monitoring respiratory symptoms, fever, flu, and diarrhea reported by emergency department records from 38 hospitals in the city. Soon the system was routinely picking up patterns that were eluding frontline clinicians, including an outbreak of highly contagious norovirus.

## Figures and Tables

**Figure:**